# Specific Immunotherapy in a Pollen-Allergic Patient With Human Immunodeficiency Virus Infection

**DOI:** 10.1097/WOX.0b013e31819bcae7

**Published:** 2009-04-15

**Authors:** Urs C Steiner, Hansjakob Furrer, Arthur Helbling

**Affiliations:** 1 Division of Allergology, University Clinic for Rheumatology and Clinical Immunology/Allergology, University Hospital (Inselspital), Bern, Switzerland; 2 Department of Infectious Diseases, Inselspital, Bern University Hospital, and University of Bern, Bern, Switzerland; 3 Division of Allergology and Clinical Immunology, University Hospital (Inselspital), 3010 Bern, Switzerland

**Keywords:** human immunodeficiency virus, pollen allergy, respiratory allergy, allergy treatment, specific immunotherapy

## Abstract

**Background**

According to the World Health Organization position paper, immunodeficiency such as human immunodeficiency virus (HIV) infection is a relative contraindication for specific immunotherapy (SIT). Since the introduction of highly active antiretroviral therapy, a significant reconstitution of immune competence in individuals with HIV is possible.

**Case Report**

In a 52-year-old man, HIV infection was diagnosed in 1987. Antiretroviral therapy was started in 1998. He presented himself in July 2001 because of an increasingly severe seasonal rhinoconjunctivitis. Symptoms were not sufficiently alleviated by various antiallergic drugs.

**Results**

The investigations showed a relevant sensitization to tree pollens. Specific immunotherapy with a tree pollen mix (hazel, birch, ash, and alder, 25% each) was started in November 2001. Viral load at this time was less than 50 copies/mL, the CD4^+ ^cell count was 307/*μ*L. Therapy was given in monthly intervals until mid-April 2005 without any side effects. Viral load and CD4^+ ^cell counts did not change during SIT. Clinically, rhinoconjunctivitis was experienced only intermittently and symptom relief was almost 90%.

**Conclusions**

This report indicates that in patients with well-controlled HIV infection on highly active antiretroviral therapy, SIT with pollen extracts is a potential and successful therapeutic option.

## 

The incidence of atopy and respiratory allergies to common inhalants in human immunodeficiency virus (HIV) -infected patients is similar as in the general population [1]. Although pollen allergy with classical rhinoconjunctivitis is not a life-threatening disease, symptoms may severely affect an individual and impair his quality of life. Therapeutic approaches are avoidance, symptomatic medications, and specific immunotherapy (SIT) with certain allergens. Often, symptomatic treatment with oral antihistamines, mast cell stabilizers, and topical corticosteroids is not sufficient to suppress clinical symptoms, and thus, allergen SIT is a potential and established option to treat inhalant allergy.

Guidelines and position papers may help in the decision making of a management. According to the World Health Organization recommendation on allergen immunotherapy, immunodeficiency such as HIV is a relative contraindication for allergen SIT [2]. However, principles do not satisfy all clinical real-life situations. Since the introduction of highly active antiretroviral therapy (HAART),[3] a significant reconstitution of immune competence in individuals with HIV is possible [4,5].

In this case report, we aim to consider SIT as a real and potential therapeutic option in HIV-positive patients if antiretroviral therapy is well established and HIV infection is controlled.

## Case reports

In a 52-year-old white man, HIV infection was diagnosed in 1987, and HAART was started in 1998 when the CD4^+ ^cell count, measured by flow cytometry, was 179/*μ*L, and the viral load was 142,412 HIV RNA copies/mL (AMPLICOR HIV-1 Monitor Test, v1.5, Roche Diagnostics, Rotkreuz, Switzerland).

He presented himself at the allergy outpatient clinic in July 2001 because he increasingly experienced a severe seasonal rhino-conjunctivitis since 1997. Symptoms were not sufficiently alleviated by various antiallergic drugs.

The investigations with skin prick tests including common aeroallergens (ALK-Abello, Horsholm, Denmark) showed relevant sensitizations to tree pollens (hazel, alder, birch, and ash). Specific immunoglobulin E (IgE) to rBet v 1 was elevated with 25.2 kU/L (class 4).

Before the initiation of immunotherapy, the patient has been fully informed about the potential risks and the lack of knowledge on safety and efficacy of immunotherapy in patients with HIV infection. Viral load was less than 50 copies/mL and the CD4^+ ^cell count was 307/*μ*L before the subcutaneous SIT with hazel, birch, ash, and alder, 25% each, Alutard SQ (ALK, Denmark), was started in November 2001 using a cluster regimen.

Pretreatment was done with the antihistamine fexofenadine 180 mg 1 hour before injection. The up-dosing phase until reaching the maintenance dose of 100,000 SQ-U was uneventful. Maintenance dose was given in monthly intervals until mid-April 2005 without any side effects except local swellings at the injection site. During therapy, CD4^+ ^cell count increased and remained greater than 350 cells/*μ*L, HIV RNA level was always less than 50 copies/mL. Specific and total IgE decreased under therapy (Table 1). Clinically, after 3 years of immunotherapy, seasonal rhinoconjunctivitis was experienced only intermittently. No additional medications were used. The overall symptom relief determined by history, visual analogue scale for subjective symptoms, and medication use was almost 90%. Today, 3 years after discontinuation of immunotherapy, typical clinical symptoms of pollen allergy did not reoccur, and no antiallergic medications were needed during the last 3 pollen seasons. The HIV infection is still well controlled under HAART.

**Table 1:** Development of CD4^+ ^Cell Count, HIV RNA Level, and Specific and Total IgE During SIT

Mo/Yr	CD4^+^/*μ*L	HIV RNA copies/mL	rBet v 1 kU/L (CAP RAST class)	IgE total, kU/L
April 2001	510	< 50	25.2(4)	232
April 2002	536	< 50	15.7(3)	
July 2003	521	< 50	12.9(3)	
July 2004	660	< 50	15.3(3)	
May 2005	579	< 50	10.2(3)	37.3
June 2008	559	< 50	13.5(3)	70.1

In summary, HIV-infected patients may experience relevant IgE-mediated respiratory diseases that need more than only symptomatic treatment. This report indicates that in patients with well-controlled HIV infection on antiretroviral therapy, SIT with pollen extracts is a potential and successful therapeutic option. Specific immunotherapy still may be considered as a relative contraindication in untreated HIV-positive patients but should probably be offered more frequently to HIV-infected patients on successful antiretroviral therapy.

## Note

The material of this study is original and has not been supported by any funding sources. There are no potential or actual conflicts of interest.
